# In Vitro and In Ovo CAM Model Evaluation of Periosteum-Derived Micrografts

**DOI:** 10.3390/jfb17030148

**Published:** 2026-03-18

**Authors:** Rawan Almujaydil, Conor J. McCann, Linh Nguyen, Francesco D’Aiuto

**Affiliations:** 1 Periodontology Unit, UCL Eastman Dental Institute, London WC1E 6DE, UK; rawan.almujaydil.20@ucl.ac.uk; 2 Department of Periodontology, College of Dentistry, Qassim University, Buraydah 52571, Saudi Arabia; 3 UCL Great Ormond Street Institute of Child Health, London WC1N 1EH, UK; conor.mccann@ucl.ac.uk; 4 Division of Biomaterials and Tissue Engineering, UCL Eastman Dental Institute, London NW3 2PF, UK; 5 Charing Cross Hospital, Imperial College Healthcare NHS Trust, London W6 8RF, UK

**Keywords:** tissue engineering, chorioallantoic membrane, biocompatible materials, regeneration, periosteum, cell- and tissue-based therapy

## Abstract

Despite advances in periodontal regenerative therapies, consistent tissue regeneration remains challenging, with cells playing an essential role in successful repair. Therefore, this study tested different dental bone substitutes embedded in the chorioallantoic membrane (CAM) combined with periosteum-derived micrografts obtained using a chair-side device (Rigenera HBW system). Cell populations within the micrografts were identified and characterised via immunofluorescence and flow cytometry (CD31, CD105, CD34, CD90, CD73, and CD45). A CAM model was employed to examine the angiogenic potential of micrografts combined with bone substitutes, which were analysed through quantitative blood vessel/vascularisation assessments using the Ikosa software (2025), along with histological and immunohistochemical evaluations such as smooth muscle actin (SMA), H&E, and Masson’s trichrome staining. Statistical analysis was performed using GraphPad Prism 10. The addition of periosteum-derived micrografts resulted in angiogenic enhancement compared to the controls. Notable enhancement of total vessel area, total length, and branching points were obtained when Fisiograft^®^ (*p* = 0.0007, *p* = 0.0002, and *p* < 0.0001, respectively), New Shore^®^ (*p* = 0.0006, *p* = 0.0149, and *p* = 0.0083, respectively), and Bio-Oss^®^ (*p* = 0.0038 and *p* = 0.0010, respectively) were combined with micrografts, compared to the positive controls. The histological and immunohistochemical analyses confirmed increased vascularisation (positive staining for SMA) in the micrograft groups. Periosteum-derived micrografts represent a promising adjunct to conventional bone-grafting materials, promoting vascularisation and potentially enhancing tissue regeneration and healing outcomes.

## 1. Introduction

The periodontium is a complex structure that anchors teeth within the alveolar socket and comprises four key components: the gingiva, root cementum, periodontal ligament, and alveolar bone [1]. Periodontitis is a chronic inflammatory disease linked to a dysbiotic dental biofilm which, if left untreated, causes progressive destruction of tooth-supporting structures (ligament and alveolar bone). Despite recent advances, complete periodontal tissue regeneration using conventional therapies remains a major challenge. Stem-cell therapy, especially that involving periosteum-derived cells (PDCs), promises to improve periodontal tissue regeneration by overcoming some of the limitations of traditional corrective treatments [2].

The periosteum—the thin connective tissue layer that covers bones—exhibits remarkable osteogenic, chondrogenic, and adipogenic capabilities, which makes it highly effective for repairing maxillofacial and alveolar bone defects [3]. Indeed, it contains a high number of osteogenic precursor cells which play a crucial role in normal bone development and repair under physiological conditions [4].

Our understanding of stem cell behaviours and how to manipulate their growth has significantly advanced regenerative medicine, facilitating clinical therapies centred on tissue engineering [5]. A key part of tissue engineering is delivering progenitor cells to the wound or mobilising endogenous cells that can proliferate and differentiate to regenerate tissues [6].

Clinical evidence indicates that stem cell therapies, including those utilising PDCs, improve clinical periodontal parameters such as clinical attachment level, probing depth, and bone defect fill when compared to cell-free treatments, without major adverse effects [7,8]. Preclinical studies have also shown that PDCs can repair critical-sized bone defects within weeks and promote new bone formation by releasing growth factors and extracellular matrix components [9]. Similarly, cultured periosteal grafts have led to vertical bone increases of 3–8 mm along with reduced probing depths in periodontitis patients without adverse effects [10].

Microblade devices (such as those from Rigenera Human Brain Wave LLC, Turin, Italy) are an innovative cell therapy technique that swiftly generates autologous micrografts from various tissues, including periosteum. Such devices not only allow for the collection of viable micrografts in real-world settings (dental practice), but also enable combined treatments (i.e., in conjunction with other biomaterials). By filtering progenitor cells > 50 μm, this technology facilitates the safe and effective harvesting of autologous micrografts that are ready to use in clinical procedures [11,12]. The safety and effectiveness of these new strategies remain uncertain, and there is still a lack of preclinical evidence to support testing new innovations. Consequently, simple and rapid models could improve preclinical studies. Among these models, the chorioallantoic membrane (CAM) assay is minimally invasive and can be used for short-term studies. While it is typically used to evaluate angiogenesis, it represents a promising platform for testing biomaterials, particularly those for cell therapy [13,14]. This study aimed to comprehensively characterise periosteum-derived micrografts using multiple analytical techniques, including flow cytometry and immunofluorescence, to assess their phenotypic and functional profiles. In addition, the study compared the interaction of these cells with different bone scaffolds commonly used in periodontal regenerative treatments by employing the CAM model as an in vivo-like experimental platform.

## 2. Materials and Methods

### 2.1. In Vitro Biological Tests

#### 2.1.1. Cell Culture

Periosteum-derived micrografts were obtained from discarded periosteal tissue samples harvested from three consecutive patients who provided informed consent. The human tissue samples and clinical data were provided by the UCL/UCLH Biobank for Studying Health and Disease (REC reference: 20/YH/0088). Periosteum samples were collected from the inner layer of a routine gum flap during standard corrective periodontal procedures and were disaggregated using the Rigenera system. Briefly, a 2 mm periosteum sample was harvested and combined with 2 mL of saline solution (0.9% NaCl) and disaggregated in the device (70 r/min and 15 Ncm for 2 min). The micrograft suspension was then collected with a sterile syringe and filtered through a 70 μm cell strainer to remove debris. The cells were seeded in T-25 culture flasks (Nunc, Thermo Fisher Scientific, Waltham, MA, USA) containing α-MEM (Minimum Essential Medium Alpha; Gibco, Thermo Fisher Scientific, Waltham, MA, USA) supplemented with 10% foetal bovine serum (FBS; Gibco) and 1% penicillin. The cultures were maintained at 37 °C in a humidified 5% CO_2_ incubator (Thermo Scientific, Waltham, MA, USA). Non-adherent cells were removed after 72 h by replacing the medium. Adherent cells were expanded until they reached 80–90% confluence, with medium changes every 3 days.

#### 2.1.2. Flow Cytometry Analysis of Cell Surface Marker Expression

Mesenchymal stem cell (MSC) markers were identified using 4 × 106 cells from cultured periosteum-derived micrografts per patient, which were digested with 0.25% trypsin–EDTA (Gibco) and then suspended in FACS buffer (PBS + 2% FBS) to obtain single-cell suspensions for flow cytometry analysis. A multicolour flow cytometry panel was designed to characterise MSC populations within the micrografts. The following antibodies were used: CD34 FITC (mouse; BioLegend, San Diego, CA, USA), CD105 PE (mouse; BD Biosciences, San Jose, CA, USA), CD73 NovaFluor™ Yellow 690 (rat; Thermo Fisher Scientific, Waltham, MA, USA), CD90 APC (mouse; Thermo Fisher Scientific, Waltham, MA, USA), CD45 BV711 (rat; BD Biosciences, San Jose, CA, USA), and CD31 APC-eFluor™ 780 (mouse; Thermo Fisher Scientific, Waltham, MA, USA). Aliquots of 1 × 106 cells per patient were incubated with 20 μL of the antibody cocktail for 30 min at 4 °C in the dark. After incubation, the cells were washed twice with FACS buffer and centrifuged at 400× *g* for 5 min to remove any unbound antibodies. To exclude dead cells, DAPI (1 μg/mL) was added to the final resuspension before acquisition. To enhance compensation and controls to ensure accurate interpretation of the flow cytometry data, a comprehensive compensation matrix was established. Compensation Beads (BD Biosciences, San Jose, CA, USA) were stained with each fluorochrome used in the experiment.

#### 2.1.3. Immunofluorescence

Cells were cultured in 6-well plates (1 × 10^4^ per well) for 7 and 14 days. On the staining day, cells were fixed with 4% paraformaldehyde for 20 min, washed with PBS, treated with 0.1% Triton for 30 min, and then blocked with 5% goat serum for 1 h. The following antibodies were used: CD90 APC (mouse; Thermo Fisher Scientific, Waltham, MA, USA), CD105 FITC (mouse; BioLegend), CD73 NovaFluor™ Yellow 690 (rat; Thermo Fisher Scientific, Waltham, MA, USA), CD34 FITC (mouse; BioLegend, San Diego, CA, USA), and CD45 Alexa Fluor™ 532 (mouse; Thermo Fisher Scientific, Waltham, MA, USA). The cells were stained with fluorophore-conjugated antibodies (the same as those used for flow cytometry), followed by nuclei counterstaining with DAPI. Imaging was performed using a confocal microscope (BioRad Radiance2100, Hercules, CA, USA; Zeiss, Jena, Germany).

### 2.2. CAM Assay

Micrografts were obtained using a similar method to the one described in Section 2.1.1 but were implanted directly without expansion (*n* = 3).

Biomaterials: Several clinically approved biomaterials for periodontal tissue were evaluated: Geistlich Bio-Oss^®^ (BO), Geistlich Bio-Oss Collagen^®^ (BC), Geistlich Mucograft^®^ (MS) (Geistlich Pharma, Wolhusen, Switzerland), and Fisiograft^®^ (FG) and New Shore^®^ (NS) (Human Brain Wave, Turin, Italy).

Experimental design: Five bone regenerative biomaterials (BO, BC, MS, FG, and NS) were tested for their ability to support periosteum-derived micrograft activity using the in ovo CAM assay. Each material was examined under three conditions: (1) positive control with scaffold only, (2) negative control with filter paper only, and (3) test case with scaffold plus micrografts. Each group included 24 fertilised chick embryos (total *n* = 264 embryos), which provided over 90% statistical power to detect moderate effect sizes (Cohen’s d = 0.5) at α = 0.05 with two-tailed t-tests.

CAM preparation: Fertilised Shaver Brown chicken eggs (Medeggs, UK) were incubated at 37 °C. The eggs were windowed at embryonic day (E) 1.5–2 and sealed with clear tape before incubation for an additional 4 days until E6. At E6, 100 μL of antibiotics was added to 20 mg or 2 × 2 mm segments (approx. 20 mg) of the biomaterial scaffolds. Under a stereoscope, a suitable area for CAM engraftment was selected. Ideally, this area contained two second- or third-order blood vessels (branches from the main artery) and a capillary bed between them. The engraftment material was placed onto the selected blood vessels. The eggs were sealed with clear tape and then returned to the incubator for a further 7 days (i.e., until E13). To ensure blinded outcome assessment, the eggs were coded after implantation and all subsequent image acquisition and analyses were performed blinded to the tested biomaterial. At E13, images were taken using a light microscope (CK2, Olympus, Tokyo, Japan). The CAM assay used fertilised chicken eggs at a developmental stage where they are not protected by relevant regulations; thus, formal animal ethics approval was not required. Nonetheless, relevant items from the ARRIVE 2.0 Essential 10 checklist, such as experimental design, sample size, outcome measures, and statistical analysis, were adhered to.

### 2.3. Optical Coherence Tomography (OCT)

At E13, the chick embryos were sacrificed, and a 1 × 1 cm section of the CAM containing the implanted material was removed by fine dissection, fixed with 4% PFA for 1 h, and stored in PBS. The CAM (1 × 1 cm) with scaffold was scanned using a VivoSight OCT system (Michelson Diagnostics, Kent, UK).

### 2.4. Histology and Immunohistochemistry

After OCT imaging, the samples were processed for paraffin embedding. Three-micrometre serial sections were obtained using a Leica RM2235 microtome (Leica Biosystems, Nussloch, Germany), and routine haematoxylin and eosin staining and Masson’s trichrome staining were performed for histologic evaluation. To assess angiogenesis, selected sections underwent immunohistochemistry to evaluate the expression of alpha-smooth muscle actin (Mouse, Thermo Fisher Scientific, Waltham, MA, USA). A minimum of 5 eggs was used for each group.

### 2.5. Statistical Analysis

Statistical analyses were conducted with GraphPad Prism version 10 (GraphPad Software, San Diego, CA, USA). The data were tested for normality. Differences between groups were evaluated using one-way ANOVA. Post hoc multiple comparison tests, such as Tukey’s test, were performed. A *p*-value less than 0.05 was deemed statistically significant.

## 3. Results

### 3.1. Isolation of Donor Cells from Periosteum-Derived Micrografts

**Figure 1:**
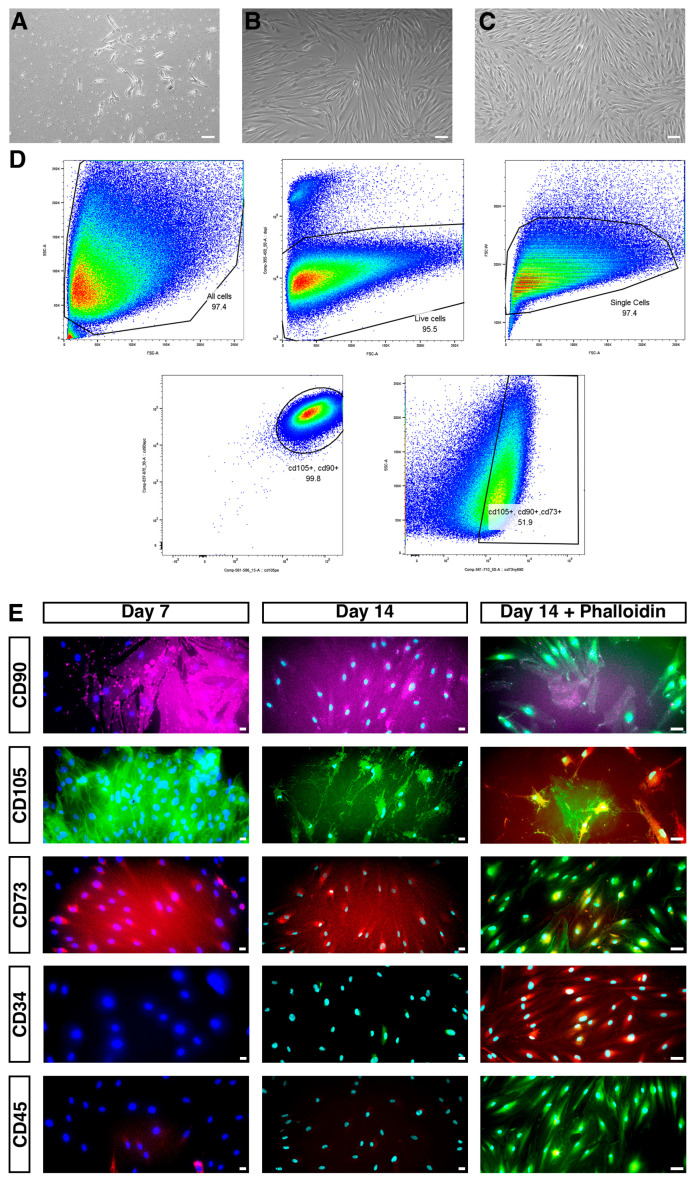
(**A**) Cells beginning to exhibit a characteristic fibroblast-like morphology, with adherence to the plastic surface and visible early monolayer outgrowths (scale bar = 40 µm). (**B**) Pre-first-passage cells growing in a confluent monolayer, indicating active proliferation and readiness for subculturing (scale bar = 10 µm). (**C**) Pre-fourth passage, cells maintain their fibroblastic properties, reflecting a sustained proliferative capacity and morphological stability across passages (scale bar = 100 µm). (**D**) Representative flow cytometry dot plot showing a triple-positive mesenchymal stem cell-like population (CD105^+^, CD90^+^, and CD73^+^) within the gated region, comprising 51.9% of analysed events. (**E**) Immunofluorescence staining confirming positivity for CD105^+^, CD90^+^, and CD73^+^ (scale bar = 20 µm).

The cultured periosteum-derived micrografts displayed the characteristic morphology of human PDCs (mononuclear, fibroblast-like, spindle-shaped, and plastic-adherent). These cells typically emerged within 5–8 days, independent of donor gender and age. Confluent cultures reached first passage after an additional 7–10 days (Figure 1A–C). Flow cytometry analysis of the periodontium-derived micrografts from three patients revealed consistent expression patterns of MSC markers and lineage-specific antigens (Table 1). All viable cells (DAPI^+^) uniformly expressed CD90^+^/CD105^+^ (100%), confirming their mesenchymal lineage origin (Table 1).

Flow cytometry analysis showed that CD45 (haematopoietic lineage marker) was minimally expressed in all samples, confirming the non-haematopoietic nature of the isolated populations. CD90 and CD105, which are MSC markers, showed robust expression in nearly all viable cells across all patient samples, indicating the presence of MSC-like subpopulations. CD73, a key marker of multipotent MSC, was expressed in almost half of the cells. Dual-positive gating for CD73^+^/CD105^+^ allowed for the identification of a functionally relevant MSC-like subset. Triple MSC marker-positive cells (CD90^+^/CD105^+^/CD73^+^) accounted for a consistent proportion (~48%) of the live population (Figure 1D). This is likely a heterogeneous periosteal stromal population containing an MSC-like subpopulation.

Immunofluorescence analysis confirmed that the cells exhibited a characteristic fibroblastic morphology with strong F-actin expression throughout the cytoplasm, confirming cytoskeletal organisation and adherence. DAPI staining showed minimal signs of nuclei fragmentation, indicating healthy cells. The high cell density and well-spread morphology observed in multiple fields indicate robust proliferation and attachment (Figure 1E).

### 3.2. Histology and Immunohistochemistry

**Figure A1:**
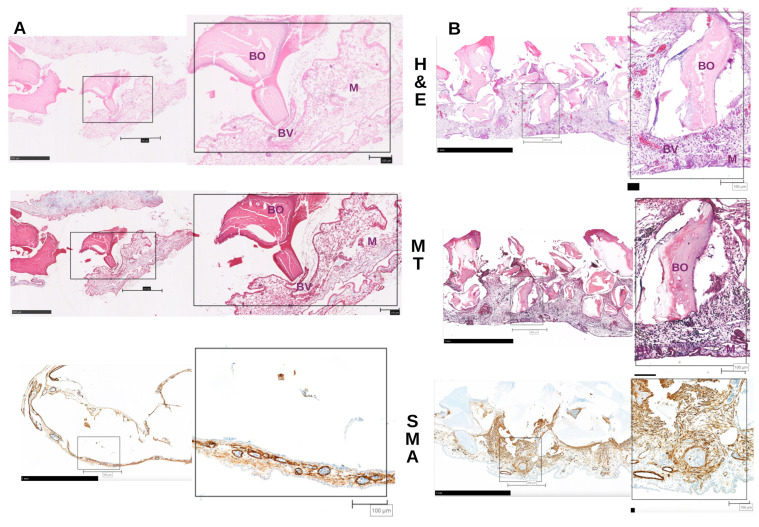
MT, H&E, and SMA staining of (**A**) BO control group showing blood vessels (BV) surrounding BO bone granules and (**B**) BO test group exhibits a higher number of blood vessels, indicated by positive brown staining, and shows increased membrane filtration between the bone granules.

**Figure A2:**
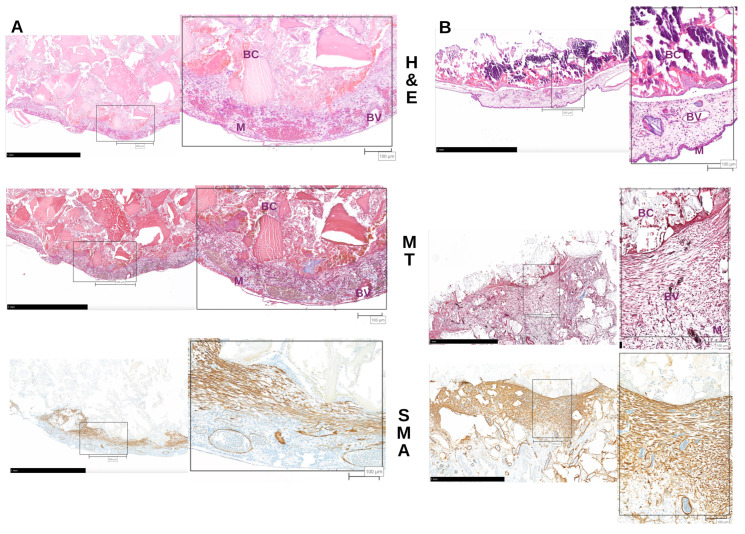
MT, H&E, and SMA staining of (**A**) BC control group showing blood vessels (BV) surrounding BC scaffold and (**B**) BC test group showing more blood vessels and positively stained tissue.

**Figure A3:**
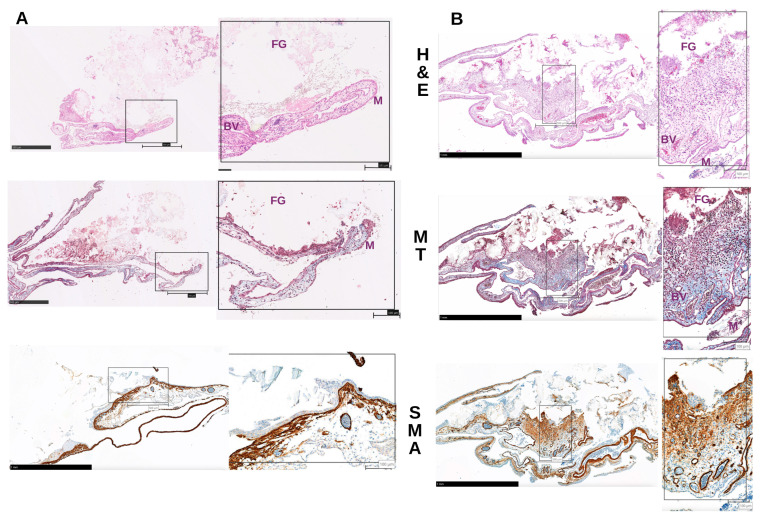
MT, H&E, and SMA staining of (**A**) FG control group showing blood vessels (BV) surrounding FG bone granules and (**B**) FG test group showing a greater number of blood vessels and greater membrane filtration between the bone granules.

**Figure A4:**
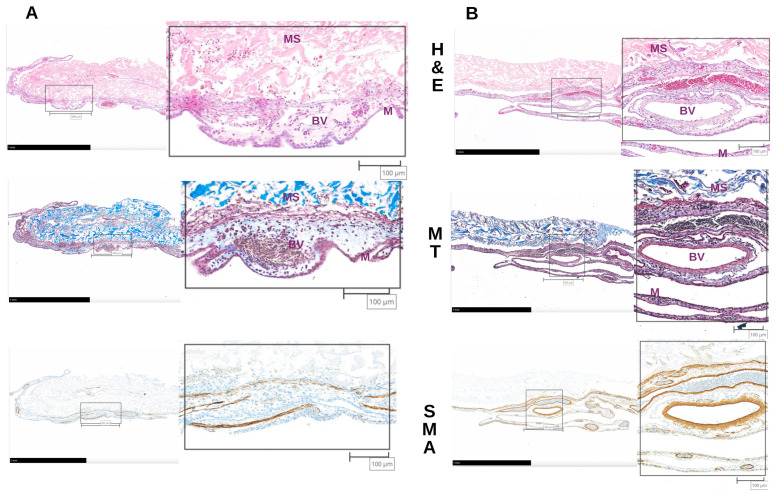
MT, H&E, and SMA staining of (**A**) MS control group showing blood vessels (BV) surrounding MS bone scaffold and (**B**) MS test group.

**Figure A5:**
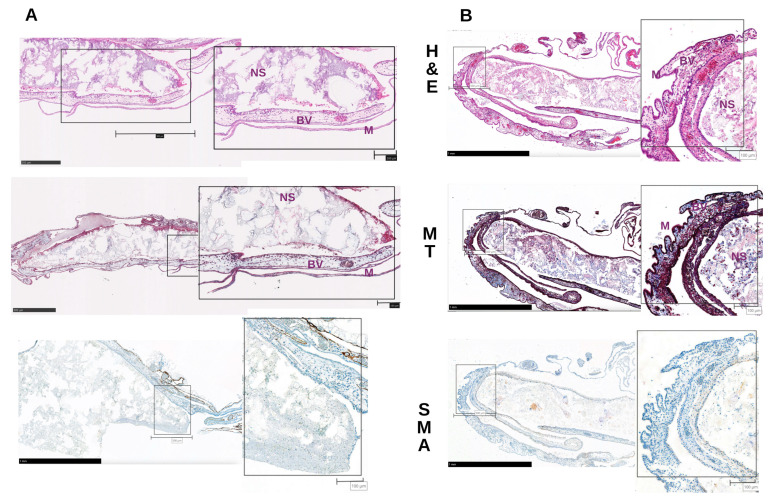
MT, H&E, and SMA staining of (**A**) NS control group showing blood vessels (BV) surrounding NS bone granules and (**B**) NS test group.

We assessed periosteum-derived micrograft engraftment using several clinically approved periodontal tissue biomaterials Geistlich Bio-Oss^®^, Geistlich Bio-Oss Collagen^®^, Geistlich Mucograft^®^ (Geistlich Pharma, Wolhusen, Switzerland), and Fisiograft^®^ and New Shore^®^ (Human Brain Wave, Turin, Italy) grown on CAM. Seven days post-seeding (E13), Bio-Oss^®^ (BO) was surrounded by host connective tissue. In the controls, the presence of blood vessels (BVs) around BO indicated moderate vascular infiltration, which was confirmed by SMA staining showing developing or mature vasculature. The test groups showed enhanced regeneration, with more interspersed vessels between bone granules and denser vascular networks, suggesting increased angiogenesis (Appendix A, Figure A1). MT, H&E, and SMA staining revealed vascular features in the Bio Oss Collagen^®^ (BC) scaffolds. The controls showed moderate vascular ingrowth with early vessel maturation signs, while the test groups showed higher vessel densities, extensive infiltration, and signs of vessel stabilisation (Appendix A, Figure A2). Fisiograft^®^ (FG) scaffolds showed initial presence of BVs with sporadic vascular smooth muscle and limited maturity in the controls. The test groups exhibited significantly more vessels, membrane-like tissue, and well-organised vessels, indicating active remodelling (Appendix A, Figure A3). The Mucograft^®^ (MS) controls displayed moderate vascular infiltration with immature vessels, while the test groups showed increased vessel density, connective tissue, and organised matrix, indicating more mature vasculature (Appendix A, Figure A4). The New Shore^®^ (NS) controls had limited organisation and moderate vascularisation, while the test groups showed increased vessel density and tissue integration, with well-vascularised zones and mature vessels (Appendix A, Figure A5).

### 3.3. Incorporation of Periosteum-Derived Micrografts Enhances Angiogenesis in Bone Substitute Materials

**Figure 2:**
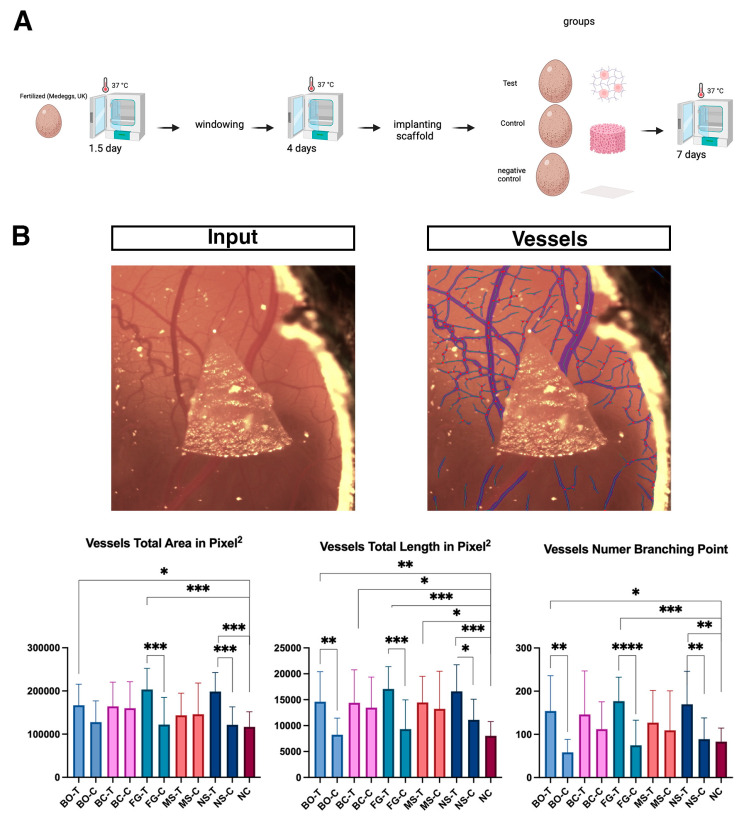
Angiogenic effects of periosteum-derived micrografts assessed via CAM assay. (**A**) CAM assay process. (**B**) Representative images of the CAM surface before (**left**) and after (**right**) vessel segmentation (lengths shown in blue, branching points shown in red, and areas shown in purple) using Ikosa software (2025), highlighting vascular networks. Graphs of the angiogenic parameters total vessel area, total vessel length, and branching points of the different treatment groups. One-way ANOVA *p* values: * *p* ≤ 0.05, ** *p* ≤ 0.01, *** *p* ≤ 0.001, **** *p* ≤ 0.000.

**Figure 3:**
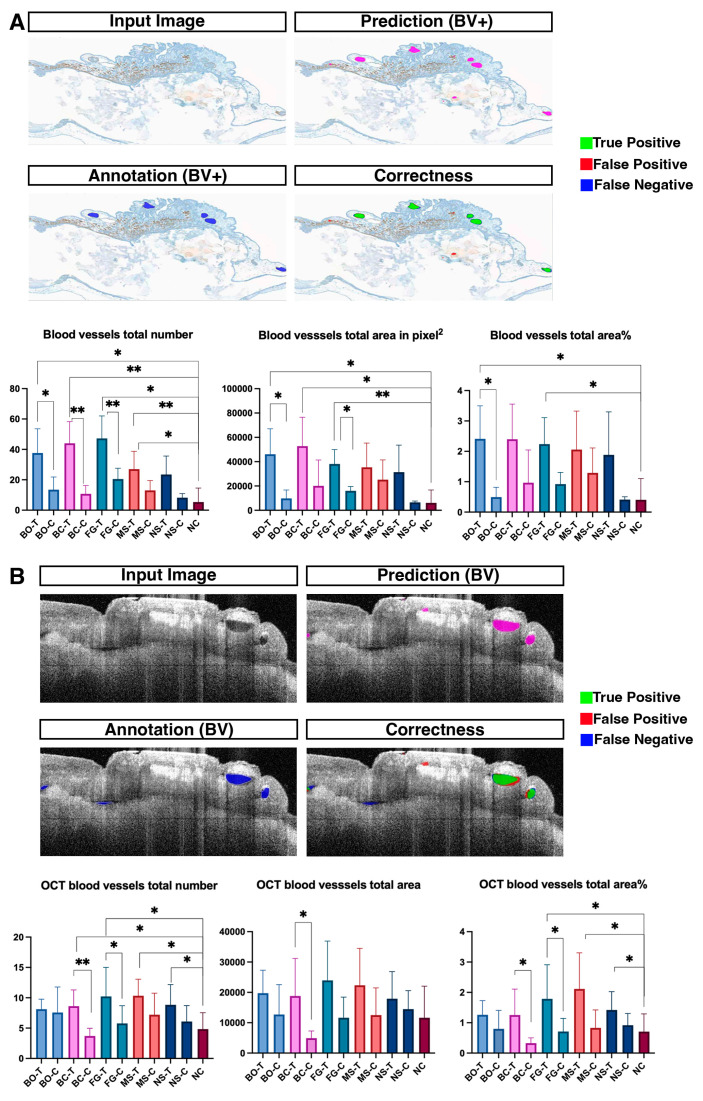
(**A**) SMA expression analysed using Ikosa AI, including a quantitative assessment of blood vessel parameters. Graphs depict the total vessel area, number, and area percentage in the different treatment groups. (**B**) OCT analysis and quantitative assessment of blood vessel parameters using the Ikosa AI software. Graphs show the total vessel area, number, and area percentage in the various treatment groups. One-way ANOVA *p* values: * *p* ≤ 0.05, ** *p* ≤ 0.01.

Survival to endpoint (E13) varied between 42 and 71% across conditions (Appendix A, Table A1). The statistical analyses only included surviving embryos (total *n* = 127 test, *n* = 124 control, *n* = 10 negative). There were no significant differences in survival between paired conditions (Fisher’s exact test, all *p* > 0.05). To assess the angiogenic properties of the periosteum-derived micrografts, we performed CAM assays. The addition of micrografts markedly enhanced angiogenesis compared to the control groups. Quantitative analysis using Ikosa confirmed that compared to Fisiograft^®^ alone, Fisiograft^®^ + micrograft resulted in a greater total vessel area (*p* = 0.0007) and total vessel length (*p* = 0.0002), and more branching points (*p* = 0.0001). Similarly, compared to New Shore^®^ alone, New Shore^®^ + micrograft showed statistically significant increases in vascular parameters: total vessel area (*p* = 0.0006), total vessel length (*p* = 0.0149), and branching points (0.0083) Lastly, Bio-Oss^®^ + micrograft vs. Bio-Oss^®^ alone showed differences in total vessel length (*p* = 0.0038) and branching points (*p* = 0.001) (Figure 2A,B). Our analysis of the OCT and SMA images using Ikosa AI revealed differences between the various micrograft groups, primarily between Fisiograft^®^ + micrograft and Fisiograft^®^ alone, as well as between Bio Oss Collagen^®^ + micrograft and Bio Oss Collagen^®^ alone, across different parameters (Figure 3A,B). These findings support a pro-angiogenic effect of periosteum-derived micrografts when combined with biomaterial scaffolds.

## 4. Discussion

Conventional periodontal regenerative procedures are associated with variable clinical outcomes including clinical failure or incomplete success due to several factors such as patient-specific characteristics (e.g., smoking, poor plaque control), surgical approach, choice of biomaterials, and insufficient surgical skill [15]. Complete regeneration of lost tissues has not been consistently achieved; therefore, cell-based therapies have recently been proposed as promising alternatives for periodontal regeneration to address these limitations [16]. Dental stem cells demonstrate potent regenerative potential when treated with biomaterials and growth factors [17,18]. In this field, periosteum-derived micrografts could provide cells to facilitate complete regeneration when combined with different biomaterial scaffolds. The efficacy of micrografts has already been demonstrated in dentistry studies, which have reported that they can enhance periodontal tissue regeneration [19]. Moreover, an in vivo study demonstrated that when applied to graft rat calvaria, micrografts resulted in improved bone regeneration. Radiographic examinations indicated a significantly greater bone volume at 1 and 2 weeks after surgery and histological analysis revealed new bone formation with lacunae of various sizes, primarily near the edges of the defect, with substantially more new bone compared to the control group [20].

In the present study, the flow cytometry findings demonstrated that periosteum-derived micrografts provide a reliable and reproducible source of a heterogeneous periosteal stromal population containing a reproducible MSC-like subpopulation with consistent expression of the canonical markers CD90 and CD105 across multiple donors. The presence of triple-positive cells (CD90^+^/CD105^+^/CD73^+^) suggested a reservoir of primitive stromal precursors that may contribute to the regenerative capacity. Notably, periosteum-derived micrografts began to migrate at day 6, as observed under a microscope at 40× magnification. Similar studies have reported the appearance of the first cellular colonies after 15–20 days [12] or from days 1 to 9 [21]. In the latter study, the cells developed into colonies and spherical clusters. The majority of the cells displayed a spindle shape and showed homogeneity under microscopic examination [21]. After 14 days of culturing, the cellular morphology remained spindle-shaped, aligning with the findings of this study. Variations in cellular morphology between micrograft studies could be due to different tissue sources, fragment sizes, devices, and donor variability, affecting cell migration and matrix degradation. Another study compared differences in culture timing between micrograft and enzymatic preparations. These differences reflect their distinct preparation mechanisms; in particular, enzymatic digestion rapidly creates a suspension of single cells capable of immediate attachment and proliferation, while micrografting preserves tissue fragments with native matrix, requiring cells to migrate and remodel before proliferating, which results in slower growth [22].

Importantly, the low expression of CD45 and CD34 confirms the non-haematopoietic origin of these cells, while the variability in CD31 expression reflects donor-specific differences or subtle contamination from vascular tissues during periosteal harvesting. Despite this, the purity and consistency of MSC markers across patients support the use of this method to generate autologous grafts for regenerative applications. Another study examining periosteum-derived micrografts reported CD90^+^ (52%), CD105^+^ (82%), and CD73^+^ (82%) expression and negative CD45 expression [12]. Additionally, another study examined human palatal periosteum stem cells, which tested CD90^+^/CD105^+^/CD73^+^. The cultured cells showed 73.0 ± 6.7% (mean ± SD) positivity for all three MSC-specific markers and exhibited a negligible presence of haematopoietic stem cell markers (0.5 ± 0.3% (mean ± SD)) [23]. The variation in the percentages of cells expressing CD105, CD90, and CD73 in studies of periosteum-derived cells could be due to various biological factors. Donor-related factors such as age, systemic health, and where the cells are taken from in the mouth can affect the number and characteristics of mesenchymal progenitors in periodontal tissues [24].

Here, we used the CAM assay to analyse blood vessel development within biomaterial scaffolds and reported our initial observations and indications of bone formation. Several techniques were used to analyse the images produced through testing of the scaffolds using the CAM model (manual or automated methods). Promising results were obtained from these novel technologies that leverage artificial intelligence methods to automate and enhance the description of vascular network structures (such as the Ikosa platform © from KML Vision GmbH) [25,26]. Importantly, the CAM features a rapidly developing vascular network that provides an ideal testing platform to examine the angiogenic properties of various donor materials for tissue regeneration [13,27,28,29]. The chick embryo has become a valuable preclinical model in dental research because it lacks a nervous system, which makes it less sensitive than other animal models. Although it is generally considered an immunodeficient host for graft implantation, some studies indicate that it can still initiate a primitive immune response [30,31].

This study is the first to use the Ikosa software to quantify the vascular network in CAM assays. Our findings indicated that this application outperformed traditional manual counting in precision when examining branching points and average vascular thickness. Moreover, it allowed for the assessment of additional vascular parameters that cannot be evaluated through manual methods [32]. To visualise the CAM assay membrane, OCT was employed to generate high-resolution cross-sectional images of inhomogeneous samples, offering a 3D representation of internal biological microstructures. This enabled optical biopsies of biological specimens [33,34,35]. Combination treatments involving periosteum-derived micrografts demonstrated a trend toward enhanced vascular remodelling compared to scaffold-only treatments. Although differences between groups were observed, some combinations, such as Fisiograft + micrograft and New Shore + micrograft, exhibited elevated values across multiple parameters, supporting the potential of micrografts in promoting vessel maturation and smooth muscle integration. Although the scaffold + micrograft group demonstrated enhanced angiogenic activity compared with the other groups, the study design did not include a micrograft-only group.

### Clinical Implications

Periosteum-derived micrografts showed considerable potential for enhancing bone tissue regeneration. They could be a valuable tool in tissue engineering treatments by providing cellular progenitor cells. In tissue engineering, the integration and safe delivery of progenitor cells with extracellular scaffolds and/or biological mediators (i.e., growth factors) is key to achieving effective and predictable clinical outcomes [36,37]. For instance, the placement of dental implants in maxillary sinus lift procedures using a combination of autologous periosteum-derived micrografts with PLGA/HA scaffolds (Alos) achieved quicker bone regeneration, greater amounts of vital mineralised tissue (54.3% compared to 44.7% in control groups), and enhanced radiographic opacity within 4 months [11]. This approach leverages the periosteum’s innate osteogenic properties, which are attributed to its progenitor cells’ high proliferation rates and multipotency, even in older patients [38,39,40]. The clinical benefits of micrografts include lower donor site morbidity, decreased immune rejection (due to their autologous origin), and compatibility with biomaterial scaffolds to address larger defects [41,42]. However, further research is needed to refine protocols for harvesting, scaffold integration, and growth factor delivery to ensure their therapeutic efficacy across various clinical contexts.

## 5. Conclusions

Micrografts derived from periosteum, when combined with various periodontal grafting materials, significantly enhance vascularisation.

## Figures and Tables

**Table 1:** Marker expression in patients.

Marker	Mean ± SD
Viable Cells	95.35 ± 1.25
CD90^+^	100 ± 0
CD105^+^	100 ± 0
CD73^+^	48.2 ± 3.7
CD31^+^	60.8 ± 32.8
CD34^+^	0.4 ± 0.2
CD45^+^	0.15 ± 0.0

## Data Availability

The datasets generated and analysed during the current study are available from the corresponding author on reasonable request.

## Appendix A

**Table A1:** CAM model survival using different biomaterials.

Biomaterial	Survival
BOtest vs. BOcontrol	14/24 (58%) vs. 17/24 (71%)
BCtest vs. BCcontrol	13/24 (54%) vs. 13/24 (54%)
MStest vs. MScontrol	11/24 (46%) vs. 12/24 (50%)
FGtest vs. FGcontrol	15/24 (63%) vs. 12/24 (50%)
NStest vs. NScontrol	10/24 (42%) vs. 10/24 (42%)
Negative control	10/24 (42%)
